# Supplementary material for: Effect of Media Composition and Oxygen Tension on Cellular Stress Response and Nrf2 Activation in HepG2ARE Cells
Source: Antioxidants (Basel). 2025 Jan 24;14(2):137. doi: 10.3390/antiox14020137 (PMC11851573; doi:10.3390/antiox14020137)
Supplement: Supplementary file 1 [file antioxidants-14-00137-s001.zip › antioxidants-3407872-supplementary.pdf]

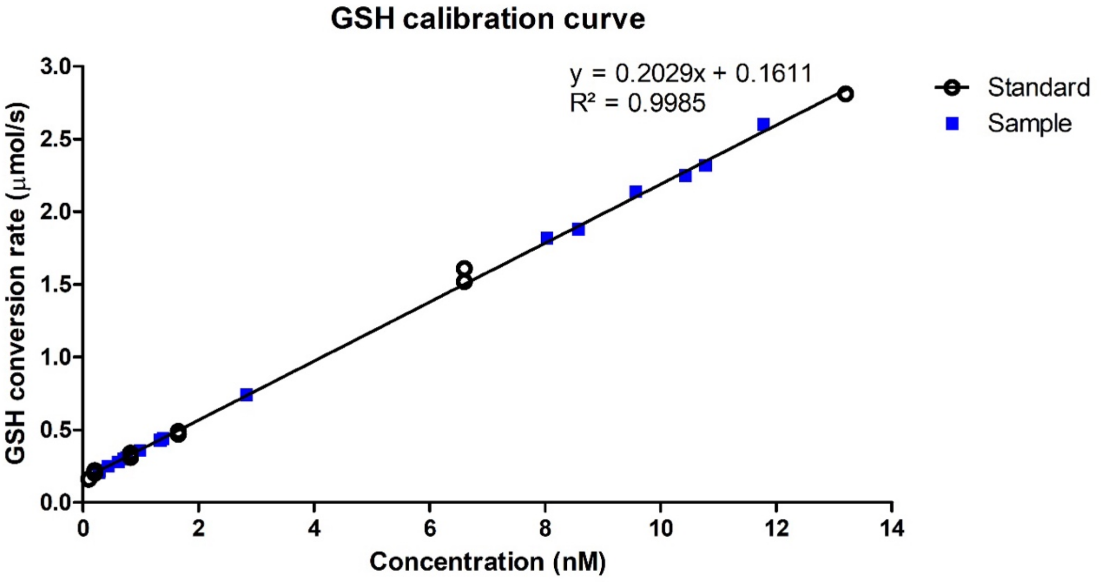

Figure S1. Calibration curve to determine the amount of GSH in HepG2ARE cell extracts. The GSH standard (0,103 nM – 13,2 nM) is black, and blue squares show some representative samples. A short explanation of the method is that GSH reacts with DTNB, producing a TNB chromophore (GS-TNB adduct). The rate of formation of TNB is proportional to the concentration of GSH in the sample. Standard and samples were measured spectrophotometrically at 412 nm every 20 seconds for 2 minutes. Absorbance values vs. time were plotted and fitted a linear regression line. 1/slope was found for each standard concentration and sample from linear regression. 1000*slope was calculated, and these values were plotted against standard concentrations. Unknown sample concentrations were interpolated from 1000*slope.

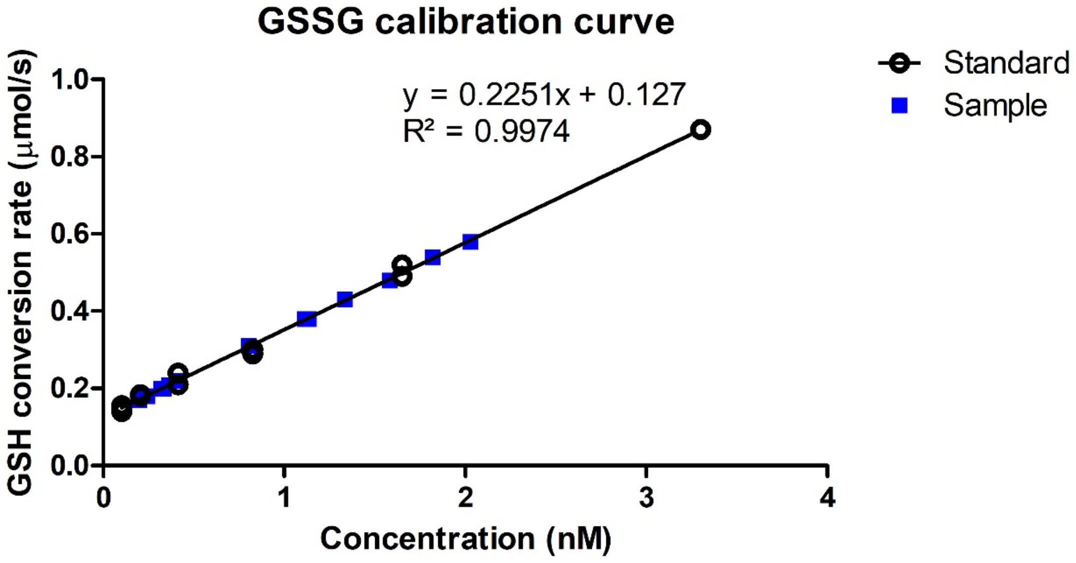

Figure S2. Calibration curve for measuring the amount of GSSG in HepG2ARE cell extracts. The GSH standard (0,103 nM – 3,3 nM) is shown in black, and a selection of samples in blue. Briefly - GSSG was reduced to GSH by glutathione reductase (GR), producing 2GSH, which reacts with DTNB and can be measured spectrophotometrically at 412 nm. Acquired values were thus divided by 2 to get the GSSG concentration. Standard and samples were measured every 20 seconds for 2 minutes. Absorbance values vs. time were plotted and fitted a linear regression line. 1/slope was found for each standard concentration and sample from linear regression. 1000*slope was calculated, and these values were plotted against standard concentrations. Unknown sample concentrations were interpolated from 1000*slope.

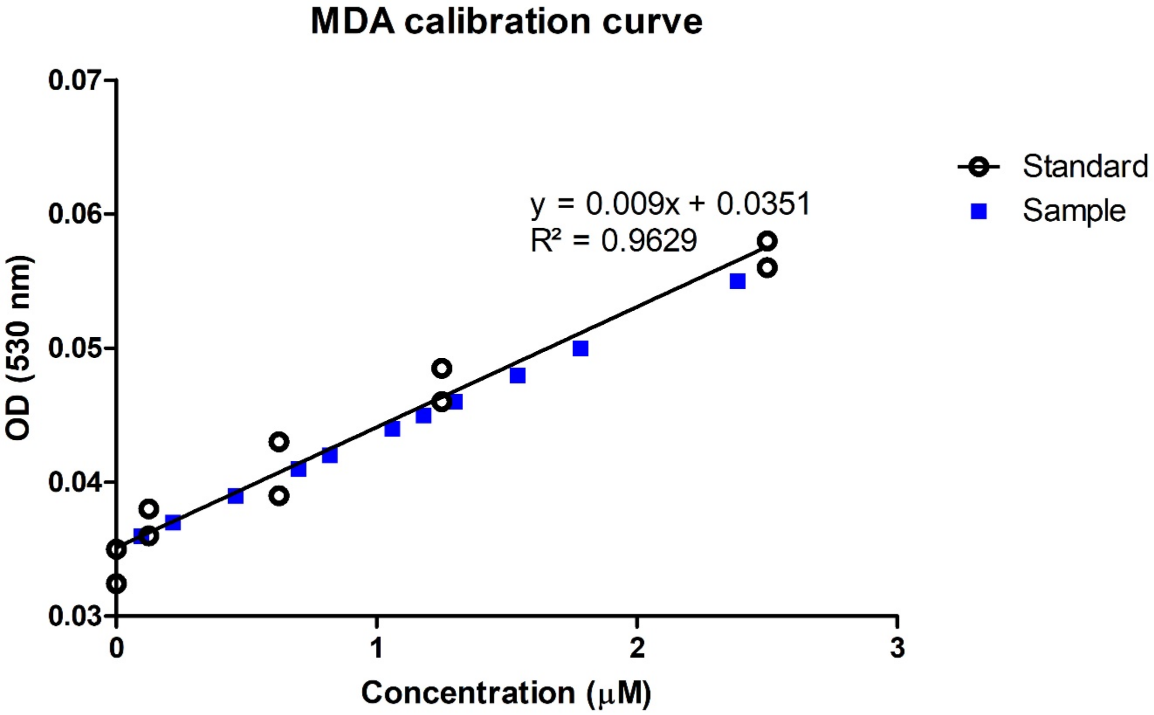

Figure S3. Calibration curve for MDA measurement from HepG2ARE cell extracts. MDA standard (0 – 2,5 μM) is depicted in black, and representative samples are shown in blue. MDA levels were detected using colorimetric Thiobarbituric Acid Reactive Substances (TBARS) assay; absorbance was measured at 530nm, and sample MDA values were calculated from the standard curve.
